# Inverse design of glass structure with deep graph neural networks

**DOI:** 10.1038/s41467-021-25490-x

**Published:** 2021-09-09

**Authors:** Qi Wang, Longfei Zhang

**Affiliations:** 1 Science and Technology on Surface Physics and Chemistry Laboratory, Jiangyou, Sichuan China; 2 School of Software, Beihang University, Beijing, China

**Keywords:** Structure of solids and liquids, Glasses, Metals and alloys, Theory and computation

## Abstract

Directly manipulating the atomic structure to achieve a specific property is a long pursuit in the field of materials. However, hindered by the disordered, non-prototypical glass structure and the complex interplay between structure and property, such inverse design is dauntingly hard for glasses. Here, combining two cutting-edge techniques, graph neural networks and swap Monte Carlo, we develop a data-driven, property-oriented inverse design route that managed to improve the plastic resistance of Cu-Zr metallic glasses in a controllable way. Swap Monte Carlo, as a sampler, effectively explores the glass landscape, and graph neural networks, with high regression accuracy in predicting the plastic resistance, serves as a decider to guide the search in configuration space. Via an unconventional strengthening mechanism, a geometrically ultra-stable yet energetically meta-stable state is unraveled, contrary to the common belief that the higher the energy, the lower the plastic resistance. This demonstrates a vast configuration space that can be easily overlooked by conventional atomistic simulations. The data-driven techniques, structural search methods and optimization algorithms consolidate to form a toolbox, paving a new way to the design of glassy materials.

## Abstract (web summary)

The inverse design of the material for given target property is challenging for glasses due to their disordered non-prototypical structure. Wang and Zhang propose a data-driven property oriented inverse approach for design of glassy materials with desired functionalities.

## Introduction

Tailoring the structure for a targeted property is a long-term pursuit of materials^1–8^. This can be formatted as an “inverse design” problem, that is, “given a target property, design the material”^1^. However, such a goal is extremely hard for glasses. Without well-defined crystal prototypes to guide the structural search, the disordered and heterogeneous structure of glasses is difficult to manipulate and design. Processing protocols such as quenching rate are usually employed to modify the glass structure, in both simulations and experiments, resulting in some control of glass properties^9^. For example, it is believed that slow quenching reduces the frozen-in excess volume and soft spots in glasses, elevating strength^10^; rejuvenation to a higher-energy state increases deformability, yet in some sacrifice of strength^11^. However, the structural regime visited by such techniques is only a small subset of the astronomic number of glass configurations that can in principle be realized. Recently, swap Monte Carlo (MC) offers an elegant way to search the configuration space, by using energy as the stability measure, and has been successfully used to simulate ultra-slow-quenched polydisperse glasses, even close to the experimental conditions^12^. However, taking energy as the decision metric naturally rules out the vast space of meta-stable configurations, which, indeed, are very likely to be obtained in experiments^9^. In addition, energy is still an indirect metric to measure the glass properties (such as the deformation resistance focused in this work), while these properties are crucial for the practical application of glasses. At present, a property-oriented inverse design protocol, namely directly designing the glass structure to optimize a specific property, is still absent.

To realize such property-oriented inverse design, typically two parts are required: a sampler, which effectively searches the vast configuration space of glasses and proposes new trial configurations, and a decider, or say forward-solver^5^, which evaluates whether a trial is acceptable and ideally guides the configuration search. As stated above, swap MC and its modified versions can fulfill the role of the sampler. However, designing an appropriate decider is dauntingly hard for glasses. The basic premises of a decider are that i) it should be accurate enough in mapping the glass structure to the property of interest, ii) it ideally should be time-efficient, as numerous structural candidates will be evaluated during the design procedure. The advent of machine learning sheds light on this avenue, with successes in correlating the static glass structure with the propensity of plastic deformation^13,14^, atomic hopping^15^ or thermal activation^16^, with computational cost orders of magnitude lower than atomistic simulations. Particularly, the renaissance of deep learning has pushed the limits of prediction accuracy in many domains^17–25^ and can serve as a better choice of decider. The powerful parallel computation capability of GPU, in terms of hardware, can also greatly boost the evaluation efficiency, to an unprecedented level.

In this work, combining two state-of-the-art techniques, namely deep graph neural networks (GNNs) as a decider and swap MC as a sampler, we have realized the inverse design of Cu–Zr metallic glasses (MGs), using the resistance to plastic deformation as an example target property. Graphs are universal models of objects and their pairwise relationships, and numerous structures are be viewed as graphs, including social networks^21^, protein interactions^22^, organic molecules^23^, and crystals^24,25^. GNN is a deep learning-based scheme that operates on graphs^26^: at each layer, information from neighbourhood is aggregated for each node (i.e., atom), and after stacking multiple layers, GNN benefits from the recursive neighborhood expansion, gradually compressing the structural information into low-dimensional atomic embeddings. A recent work from Bapst et al.^20^ has achieved impressive results using GNN to predict the long-time evolution and deformation susceptibility of Lennard-Jones liquids and glasses. As the application of GNN in the glass research is still in its infancy, there is a huge exploration space in the architecture design and application scenario. In this work, we design a different GNN framework that incorporates multi-head attention, allows for hierarchical structural information extraction in various layers, and is perfectly rotationally equivariant by encoding the interatomic distance instead of relative positions. Further incorporating GNN and swap MC, we develop an inverse design route that has managed to improve the deformation resistance of typical Cu–Zr glasses with no human intervention. An unconventional strengthening route is uncovered, with a small degree of energy sacrifice, yet achieving a remarkable gain in strengthening. We also design several strategies to accelerate the optimization process. To our best knowledge, such property-oriented inverse design of glass structure has not been reported before. It can open new avenues for the controllable manipulation of glass structure to reach a target property. The inverse design framework is general and can be conceivably applied to any property of glasses.

## Results

### GNN architecture

Unlike images (i.e., Euclidean graphs), non-Euclidean graphs, such as the glass structure here, are difficult to express and represent. Typically, key processes of GNNs are aggregation, pooling, and readout^26^. In each layer, a node aggregates information of its neighbors and itself by graph convolution and pooling, and updates its node and bond features for the next layer of message passing. After *N* layers, each node will aggregate information up to its hop-*N* neighborhoods. Finally, the node feature vectors can be fed to a readout layer for an upstream task (e.g., node classification or regression).

**Fig. 1:**
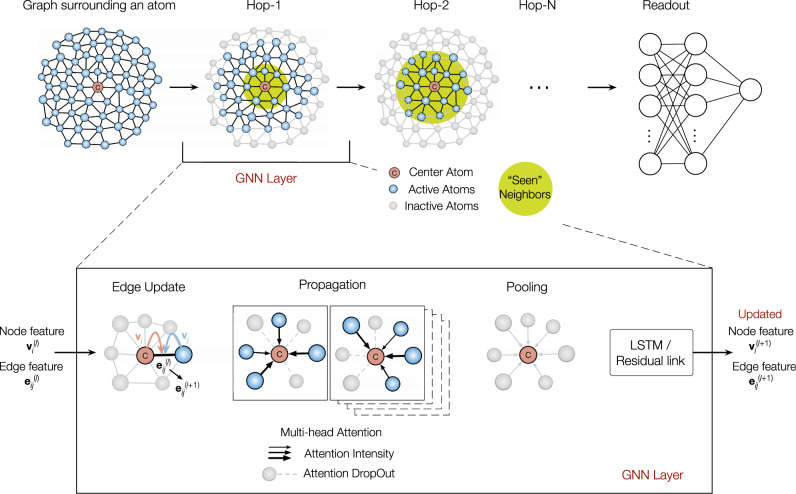
Architecture of graph neural networks. Starting from the glass graph with nodes as atoms and edges as interatomic interactions, the graph networks are stacked with *N* layers, allowing messages passing through hop-*N* neighboring layers around each atom. Finally, a readout layer is set up to get the property (regression) or the possibility of belonging to a class (classification). The processing procedure inside a GNN layer is highlighted in the lower panel.

The schematic picture of our GNN architecture is presented in Fig. 1. Specifically, the graph input is composed of a set of nodes (or vertices) and edges (or bonds), *V* = {**v**_*i*_}, *E* = {**e**_*ij*_}, where *V* is the set of nodes, **v**_*i*_ is the initial feature vector of node *i*; *E* is the set of edges, **e**_*ij*_ is the initial edge feature vectors between node *i* and *j*. Here, the initial **v**_*i*_ is just a one-hot vector of element types, e.g., [1, 0] for Cu and [0, 1] for Zr, without any hand-tuned features included. The initial **e**_*ij*_ is a vector of Gaussian expanded distance between nodes.

As illustrated in the lower panel of Fig. 1, in each layer, we first update the edge feature vectors,

1 $${{{{{{\bf{e}}}}}}}_{{ij}}^{(l+1)}={{{{{\rm{\sigma }}}}}}({{{{{{\bf{e}}}}}}}_{ij}^{(l)}+{{{{{{\bf{W}}}}}}}_{e}^{(l+1)}({{{\bf{h}}}}_{i}^{(l)}\Vert {{{\bf{h}}}}_{j}^{(l)}\Vert {{{\bf{e}}}}_{{{{{ij}}}}}^{(l)}))$$

where ${{{{{{\bf{e}}}}}}}_{{{{{ij}}}}}^{(l)}$ is the edge feature at the *l*-th layer. ${{{{{{\bf{W}}}}}}}_{e}^{(l+1)}$ is the edge linear transformation’s weight matrix. ${{{\bf{h}}}}_{i}^{(l)}$ is the hidden state of node *i*. || denotes vector concatenation. *σ* is an activation function such as sigmoid, softplus, relu, or leaky-relu, and we use softplus as default. The residual link^27^ is used to connect the output of a layer with its reference input. It makes the layer only learn the additive residual function, which is easier to learn than the original unreferenced function.

Next, we update the node hidden state in two steps. First, we calculate the local hidden state of each node using a multi-head attention scheme^28,29^. For a *k*th attention head, we calculate the interactive attention between node *i* and its neighbor *j* (Eq. 2), and then update the hidden state between *i* and *j* (Eq. 3):

2 $${a}_{ij}^{(l+1,k)}={{{{{\rm{Softmax}}}}}}({{{{{\bf{W}}}}}}_{a}^{(l+1,k)}({{{\bf{h}}}}_{i}^{(l)}\Vert {{{\bf{h}}}}_{j}^{(l)}\Vert {{{\bf{e}}}}_{{{{{ij}}}}}^{({{{l}}}+1)}))$$

3 $${{{\bf{h}}}}_{ij}^{(l+1,k)}=\sigma \big({a}_{{{{ij}}}}^{(l+1,k)}\odot ({{{{{\bf{W}}}}}}_{h}^{(l+1,k)}{{{{{\rm{Dropout}}}}}}({{{\bf{h}}}}_{i}^{(l)}\Vert {{{\bf{h}}}}_{j}^{(l)}\Vert {{{\bf{e}}}}_{ij}^{(l+1)}))$$

${{{{{\bf{W}}}}}}_{a}^{(l+1,k)}$ is the weight matrix of the *k*th attention head. The softmax function normalizes an input vector to a probability distribution, i.e., the attention weights. ${{{{{\bf{W}}}}}}_{h}^{(l+1,k)}$ is the hidden state weight matrix. ⊙ denotes the element-wise product. We also use the dropout method^30^ to let each head focus on a different representation subspace. For each node *i*, we represent all the hidden states, ${{{\bf{h}}}}_{ij}^{(l+1,k)}$, between *i* and all its neighbors *j*, from each attention head *k*.

In the second step, we concatenate all heads of hidden states, pool it, and use a remember function (abbreviated as RF in Eq. 4) to include the hidden state of the previous layer:

4 $${{{\bf{h}}}}_{i}^{(l+1)}={{{{{\rm{RF}}}}}}\left(\mathop{{{{{{\rm{Pool}}}}}}}\limits_{j\in {N}_{i}}\left({\mathop{||}_{k=1}^{{K}}}{{{\bf{h}}}}_{ij}^{(l+1,k)}\right)\right)$$

where *K* is the head number at the *l*th layer. ${\mathop{||}\limits_{k=1}^{{K}}}$means concatenating all heads of ${{{\bf{h}}}}_{ij}^{(l+1,k)}$. *N*_*i*_ is the neighborhood of node *i*. Pool indicates the pooling method to get the final hidden state of the node. We have tried set2set^31^, mean-pooling, max-pooling, and sum-pooling, and sum-pooling is found to be both robust and efficient. We also try three remember functions, namely LSTM^32,33^, dense-net^34^, and residual link^27^, and they are found to perform similarly. In accordance with the edge update function (Eq. 1), we use the residual link at default.

After *N* layers, the node features will be updated by selectively aggregating structural input from up to hop-*N* neighborhoods. Finally, we use a multi-layer perceptron with dropout as a readout layer to do the node regression. The number of tunable parameters in our GNN model is 131,274. These GNN operations are able to operate on graphs of arbitrary shape and graph size. Besides, we also implement the minibatch forward and backward propagation method on graphs^21^ to allow for learning large graphs (e.g., containing >10^5^ atoms), which would otherwise be easily out of GPU memory. For small-size graphs here (each of 16,000 atoms, to be detailed later), using the entire graph as a minibatch is better. The Pytorch^35^ implementation of our GNN model is made public in GitHub (https://github.com/Qi-max/gnn-for-glass).

In liquids and glasses, the nearest-neighbors (or say hop-1 neighbors) are frequently studied, in the context of short-range order (SRO)^36^. However, the effects of second-nearest neighbors (hop-2) and beyond, or say medium-range order (MRO), are also important but are much harder to encode. GNN offers a natural way to aggregate information from the expanded neighborhoods. This can open new possibilities for understanding the atomic structure of glasses and establishing unprecedentedly accurate structure-property relations. Our implementation is different from that of Bapst et al.^20^ in several aspects: (i) we implement multi-head attention to distinguish and quantify neighbor contribution, and the interpretability of GNN is also improved; (ii) we introduce edge dropout to perturb the graph connection in each training epoch, which can be regarded as graph data augmentation and improves the robustness of GNN training; (iii) instead of the recurrent-style implementation, we let parameters in the multiple graph layers to vary, allowing for the hierarchical structural information extraction in different stages; (iv) our GNN framework directly encodes the interatomic distances, which makes it perfectly rotationally equivariant, eliminating the need of data augmentation by adding configurations with varying orientation. In the following, we will demonstrate that our GNN framework achieves remarkable regression accuracy in predicting the plastic susceptibility in Cu–Zr MGs of different compositions and processing histories, and well fulfills the role of a decider in the later inverse design experiment.

### GNN predicting atomic-scale plastic susceptibility

In this work, we employ the plastic resistance as an example target property and train our GNN to predict the plastic propensity of atoms, from the static, unstrained structure. The models obtained will then serve as a decider for the design of glasses that are ultra-resistant to plastic rearrangement. As we’d like to the glass structure to be strengthened in a global manner, rather than merely be strengthened under a specific loading mode or direction, we lead our GNN to learn to predict a more comprehensive deformation propensity. Specifically, we stimulate twelve athermal quasi-static (AQS)^37^ loading conditions using molecular dynamics (MD) simulations and average the ln(${D}_{{{\min }}}^{2}$) as the deformation indicator of each strain, i.e., the regression target of our GNN (simulation details in Methods). We quench 120 configurations, each of 16,000 atoms, of Cu_50_Zr_50_ and Cu_64_Zr_36_ under a typical rate of 10^10^ K s^−1^. The configurations of each glass are divided into three parts: 100 configurations for training (i.e., a total of 1.6 million atomic environments), 10 for validation, and 10 for the test. GNN models are trained on the training configurations, and models with the lowest validation error are selected. Finally, the test score is derived. Note that the test set is totally unseen and does not participate in model selection. We also simulate 10 Cu_50_Zr_50_ and Cu_64_Zr_36_ configurations under a slower quenching rate of 10^9^ K s^−1^, to test the generalizability of our GNN models between different quenching rates or/and compositions. Pearson correlation coefficient is used as the major scoring metric (also for ease of comparison with previous works^20^). We allow the GNN model to train for ~5000 epochs, and in practice, the model usually reaches the best validation score at ~1000 epochs.

**Fig. 2:**
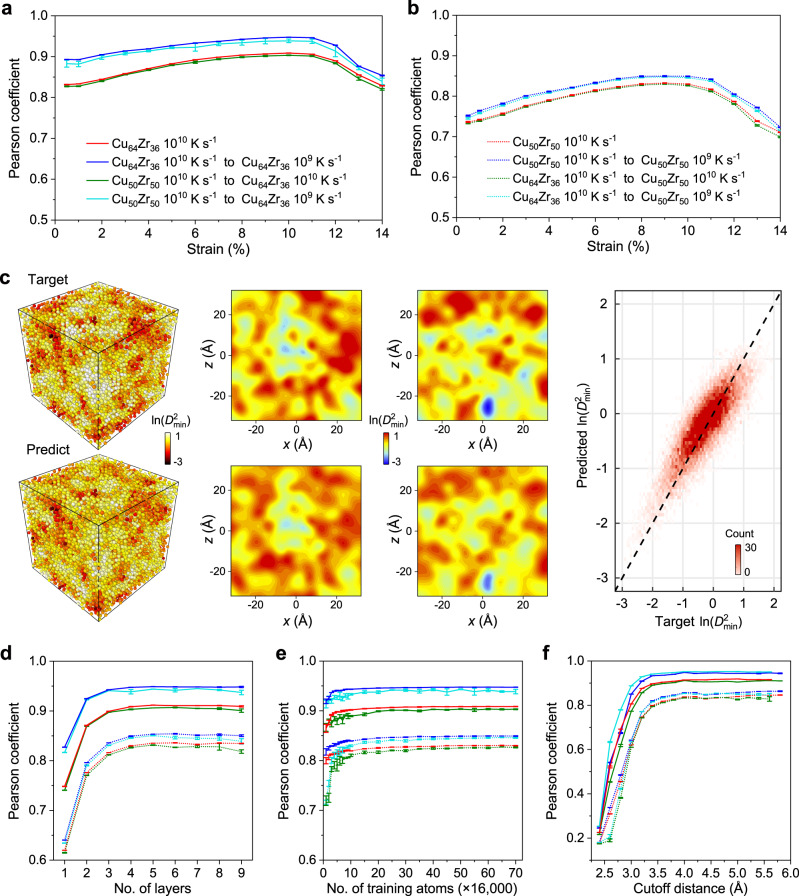
Predicting the plastic propensity using GNN. **a**–**b** Pearson coefficient of ln(${D}_{{{\min }}}^{2}$) prediction for each strain of (**a**) Cu_64_Zr_36_ and (**b**) Cu_50_Zr_50_, respectively. Each point and the error bar show the average and standard deviation of an ensemble of ten independently trained GNN models. The GNN generalization results between quenching rates or/and compositions are also presented. **c** Target (simulated, upper row) versus GNN predicted (lower row) ln(${D}_{{{\min }}}^{2}$) of a Cu_50_Zr_50_ – 10^9^ K s^−1^ configuration at strain 10%. Linear interpolation of ln(${D}_{{{\min }}}^{2}$) in typical slices of 3 Å thickness are compared in the middle panel (more slices of different strains, compositions, and quenching rates in Supplementary Figs. 1–4). The parity plot of the target and predicted ln(${D}_{{{\min }}}^{2}$) is in the right panel. **d**–**f** Pearson coefficient with varying (**d**) number of GNN layers, (**e**) number of atoms for training (derived from number of training configurations, each containing 16,000 atoms), and (**f**) cutoff distance in designating the nearest (hop-1) neighbors when building graphs around atoms.

Figure 2a, b show the Pearson coefficients of ln(${D}_{{{\min }}}^{2}$) prediction for the strains of 0.5% to 14% of Cu_64_Zr_36_ and Cu_50_Zr_50_. We find that the quality of GNN prediction keeps increasing until the strain of 10%, in the vicinity to the yielding point. This trend is similar to that of Bapst et al.^20^, which employs an “incremental” prediction scheme, i.e., using the configuration at a given tilt to predict the displacement as the tilt increases by 4%. Here, we managed to use the undeformed structure itself to predict the deformation heterogeneity at large strains, which is indeed a more difficult task. To further estimate the reliability of prediction, we independently train an ensemble of ten GNN models (bagging). Their test scores are quite close, suggesting that our training is very stable (Fig. 2a, b). For closer scrutinization, we show the target versus GNN predicted ln(${D}_{{{\min }}}^{2}$) of a Cu_50_Zr_50_ – 10^9^ K s^−1^ configuration at strain 10% (Fig. 2c). The prediction is derived by the GNN models trained and verified in Cu_50_Zr_50_ – 10^10^ K s^−1^ configurations, i.e., generalized between quenching rates, from 10^10^ K s^−1^ to 10^9^ K s^−1^. Satisfactory correspondence can be seen from the three-dimensional ln(${D}_{{{\min }}}^{2}$) distribution (left in Fig. 2c), the linear interpolation of ln(${D}_{{{\min }}}^{2}$) in typical slices of 3 Å thickness (middle, please find more slices of different strains, compositions, and quenching rates in Supplementary Figs. 1–4), as well as from the parity plot (right). We also test using the predicted ln(${D}_{{{\min }}}^{2}$) to classify the soft or hard atoms at various strains. Remarkable accuracy has been achieved, as shown in Supplementary Figs. 5–8. As an example, in the soft end, the classification metric, the area under the receiver operating characteristic curve (AUC-ROC), of Cu_50_Zr_50_ – 10^9^ K s^−1^ reaches ~0.968 under a fraction threshold *f*_thres_ of 0.5% and ~0.924 upon a much larger *f*_thres_ of 10% (Supplementary Fig. 7a). The classification performance in the hard end is even better, with AUC-ROC > 0.94 for all strains before yielding (Supplementary Fig. 7b). For Cu_64_Zr_36_ – 10^9^ K s^−1^, as another example, the AUC-ROC for soft atoms reaches ~0.983 (*f*_thres_ = 0.5%) and ~0.955 (*f*_thres_ = 10%), and the AUC-ROC for hard atoms is steadily greater than 0.98 before yielding (Supplementary Fig. 5). Besides, GNN models fitted in a single composition can also generalize well to a different composition (Fig. 2a, b), manifesting the power of GNN in representing the heterogenous atomic environments present in different glasses.

We also investigate the performance of GNN as a function of the number of graph layers (*N*_layer_), training atoms (*N*_atom_), and cutoff in defining the nearest-neighbors (*R*_c_) (Fig. 2d–f). The GNN performance gradually stabilizes at *N*_layer_ of 4 (Fig. 2d), suggesting that information up to the 4th neighbor shell is critical to the prediction. The score improves rapidly with the increase of *N*_atom_, and even ~80,000 atomic environments (equivalently, 5 configurations, each containing 16,000 atoms) are able to yield reasonable results (Fig. 2e). The low requirement of *N*_atom_ indicates an impressive learning efficiency of our GNN framework. For *R*_c_, ~4.0 Å can yield optimal results (Fig. 2f) and avoid neighborhood explosion with too large a cutoff. With the highly accurate GNN models at hand, we will proceed to demonstrate the feasibility of inverse design by integrating GNN and swap MC.

### Inverse design of deformation-resistant glass structure

**Fig. 3:**
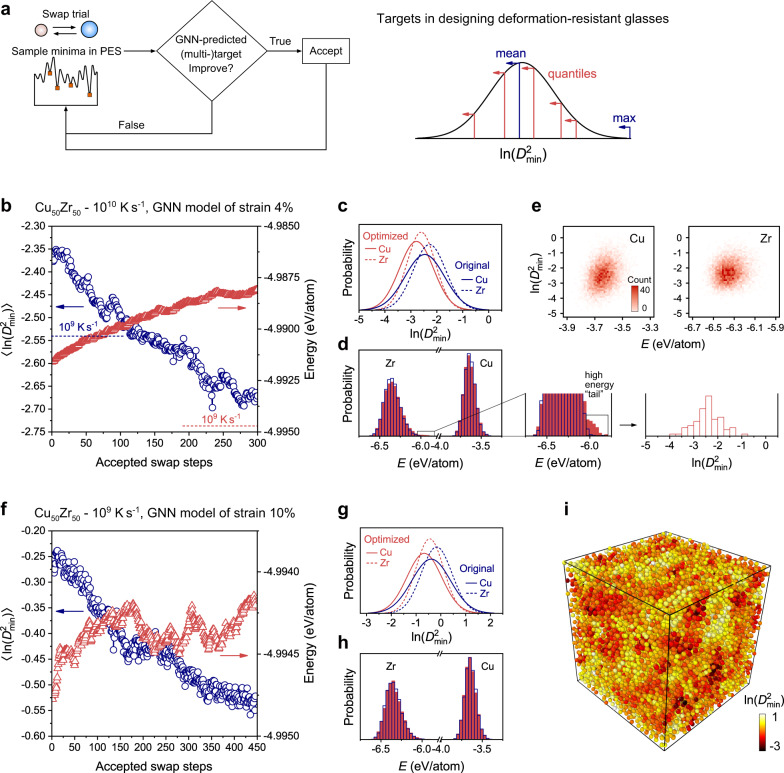
Inverse design of deformation-resistant glass structure. **a** Schematic of the iterative design scheme, based on swap MC as a sampler and GNN as a decider. The right panel shows the targets to be optimized (the maximum, mean, and quantiles) in designing the deformation-resistant glasses. **b** Evolution of configuration-average ${{{{\mathrm{ln}}}}}({D}_{{{\min }}}^{2})$ and energy, when using the GNN model for strain 4% to optimize a Cu_50_Zr_50_ − 10^10^ K s^−1^ configuration. **c** Distribution of In$({D}_{{{\min }}}^{2})$ of Cu and Zr atoms before and after optimization. **d** Distribution of energy before (blue, open histograms) and after (red, filled histograms) optimization. A high-energy tail in the energy spectra of Zr atoms is highlighted and the In$({D}_{{{\min }}}^{2})$ the distribution of these high-energy atoms are presented in the right panel. **e** Weak correlation between local energy and In$({D}_{{{\min }}}^{2})$. **f** Evolution of configuration-average ${{{{\mathrm{ln}}}}}({D}_{{{\min }}}^{2})$ and energy when using the GNN model for strain 10% to optimize a Cu_50_Zr_50_ − 10^9^ K s^−1^ configuration. **g** The distribution of In$({D}_{{{\min }}}^{2})$ before and after optimization. **h** The distribution of energy before and after optimization. **i** The simulated In$({D}_{{{\min }}}^{2})$ distribution of the optimized configuration. A systematic decrease of In$({D}_{{{\min }}}^{2})$ can be observed with comparison to the original state (“Target”, upper left in Fig. 2c).

Combining GNN and swap MC, we develop a GNN-guided, property-oriented route to strengthen model Cu_50_Zr_50_ configurations and controllably improve their resistance to plastic rearrangement. A schematic is presented in Fig. 3a and the detailed procedure is presented in Methods. Briefly speaking, in each round of trial, we first use the atom swap and energy minimization method to sample a local minimum in the potential energy surface (PES). For the sampled state, we apply GNN to decide whether this trial is acceptable or not, based on several customized target functions (e.g., right panel in Fig. 3a) and their associated acceptance criteria. The trials are iterated for multiple rounds, until the property of interest (such as the plastic resistance here) being close to the target. In practice, we also design several useful strategies to accelerate the optimization (to be described in Discussion).

We first take a Cu_50_Zr_50_ – 10^10^ K s^−1^ configuration at starting configuration and employ the GNN model for strain 4% as decider (Fig. 3b).

We tried 30,000 rounds of trials, and ~1.2% were accepted. To validate the optimization, we exhaustively simulate the AQS deformations for each accepted configuration and calculate the ln(${D}_{{{\min }}}^{2}$) at strain 4% (Fig. 3b). After ~120 accepted steps, the ln(${D}_{{{\min }}}^{2}$) of the optimized Cu_50_Zr_50_ – 10^10^ K s^−1^ configuration is already lower than that of the Cu_50_Zr_50_ – 10^9^ K s^−1^ glass (as highlighted by the blue dashed line in Fig. 3b), which is the lowest quenching rate used in this work. The detailed ln(${D}_{{{\min }}}^{2}$) distributions of Cu and Zr before and after optimization are shown in Fig. 3c. The plastic susceptibility of Cu and Zr both remarkably decreases after optimization. We note that to reduce the computation overhead and accelerate optimization, we select the GNN model with the highest validation score as decider, rather than averaging predictions from the ensemble of GNN models (whose scores are indeed similar, as observed in Fig. 2a, b).

Interestingly, if we look at the energy, the energy indeed increases during optimization (Fig. 3b). This indicates that the optimized state is not an energetically ultra-stable state, yet stands out as a “geometrically” ultra-stable state. In experiments, meta-stable states that reside in local (not global) minima of PES can still be manufactured even if they do not correspond to the thermodynamic ground state. Furthermore, despite the energy increases, the increase is merely ~3.75 meV/atom, suggesting that this state has a good chance to be sampled in real experiments. For example, according to the Boltzmann distribution, the probability of sampling an energy state is proportional to exp(−*E*/*k*_b_*T*). The smaller the energy increase, the greater the probability of sampling this meta-stable state. As another comparison, rejuvenation in experiments could even introduce ~300 meV/atom into the glass^11^. The optimized configuration has a slightly wider energy dispersion (Fig. 3d). A high-energy tail appears in the energy spectrum of Zr; however, their plastic inclination (right panel in Fig. 3d) is close to the overall distribution of Zr (Fig. 3c). This is further supported by the weak correlation between local energy and ln(${D}_{{{\min }}}^{2}$), with low Pearson correlation coefficients of 0.269 and 0.148 for Cu and Zr, respectively (Fig. 3e). The direct optimization of glass structure based on plastic resistance rather than energy as an indirect measure enables us to reveal an unconventional strengthening mechanism of glass, that is, a small energy sacrifice may help produce a remarkable gain of plastic resistance.

We further try optimizing the plastic resistance up to a larger strain of 10%, starting with a Cu_50_Zr_50_ – 10^9^ K s^−1^ configuration (Fig. 3f). The GNN model for strain 10% is used as decider. We tried 90,000 rounds of swap-displacement trials, and ~0.5% were accepted. After optimization, the ln(${D}_{{{\min }}}^{2}$) of Cu and Zr also markedly shifts to the lower end (Fig. 3g). In this case, the energy only shows a marginal increase of ~0.55 meV/atom (Fig. 3f and h), suggesting that energetic stability is more important for long-term plastic resistance than short-term resistance. The spatial distribution of ln(${D}_{{{\min }}}^{2}$) at strain 10% is derived for the optimized configuration (Fig. 3i), which has been systematically strengthened from the original state (upper left in Fig. 2c).

Given the higher energy (despite not much) of the revealed geometrically ultra-stable configurations, it is of interest to check their stability under finite temperatures and examine whether the enhanced plastic resistance can be maintained. To this end, we first anneal the two optimized configurations at the room temperature of 300 K (Supplementary Fig. 9). The ensemble is set to be canonical (NVT) to avoid the effect of volume change on plastic resistance. The introduction of temperature leads to a notable energy increase, and the system is allowed to equilibrate at 300 K for 2 ns (Supplementary Fig. 9a, b). After that, we reapply the conjugate-gradient method to get the inherent structure (this step is also essential for the later AQS deformation to examine the plastic resistance, which requires the inherent structure as starting structure). Afterwards, we perform AQS deformation simulations under the same conditions. The distribution of ln(${D}_{{{\min }}}^{2}$) for the GNN-optimized configuration, the GNN-optimized configuration after annealing at 300 K, and the original unoptimized configuration are organized in Supplementary Fig. 9a and b. It is found that after annealing at 300 K, the optimized plastic resistance can still be maintained, with a systematic strengthening from the original state. The stability of the GNN-optimized 10^9^ K/s configuration is relatively stronger than the 10^10^ K/s one. Thus, we further try annealing the 10^9^ K/s configuration at a higher annealing temperature of 600 K, which is only ~150 K below the glass transition temperature (*T*_g_). It turns out the plastic resistance can still be maintained well (lower panel in Supplementary Fig. 9b). This prominent stability can be understood in terms of the large energy barrier between the local minima of PES. Despite the optimized state has a minor energy increase from the original state, the energy barriers between these structures are indeed much larger. This enables the optimized state to well resist the imposed thermodynamic stimulus without undergoing pronounced structural change.

When the melt is quenched from high temperature, due to the drastic dynamical slowdown, it is very likely to be confined in one of the local minima in PES, rather than the global minimum. In this work, we present an interesting data-centric, property-oriented protocol to explore the glass PES. Results not only reveal an unconventional strengthening mechanism, but also indicate that even within a very small energy window and at a fixed composition, the property of glasses has a large room for adjustment. This can provide some theoretical basis for explaining the varying glass property in experiments, even under similar processing routes, and encourages further exploration of the vast and intriguing structural space of glass materials.

We believe that the optimization can go further, if we continue this optimization procedure. But it will take extra time. Our main purpose here is to demonstrate that the collaboration of GNN and swap MC is a powerful way to meet the inverse design challenge we are dealing with. Here we reveal that GNN well serves as a surrogate model to perform tests on virtual geometries and controllably explore the configuration space in search of structures with desired properties. The high accuracy of GNN makes it possible to replace expensive simulations for preselecting and optimizing the glass structures. Meanwhile, as our GNN predicts the plastic resistance at the atomic scale, we can even directionally strengthen the “weak spots” inside the glass structure instead of conducting completely stochastic trials over all atoms (to be elaborated in the Discussion section). The optimization task here is to improve the plastic resistance, and one can devise different optimization tasks by developing customized GNN models and designing customized optimization metrics. One can even use this strategy to design complex glass structures with some specific pattern of hard and soft regions for more sophisticated use-cases.

### Interpreting the unconventional strengthening

**Fig. 4:**
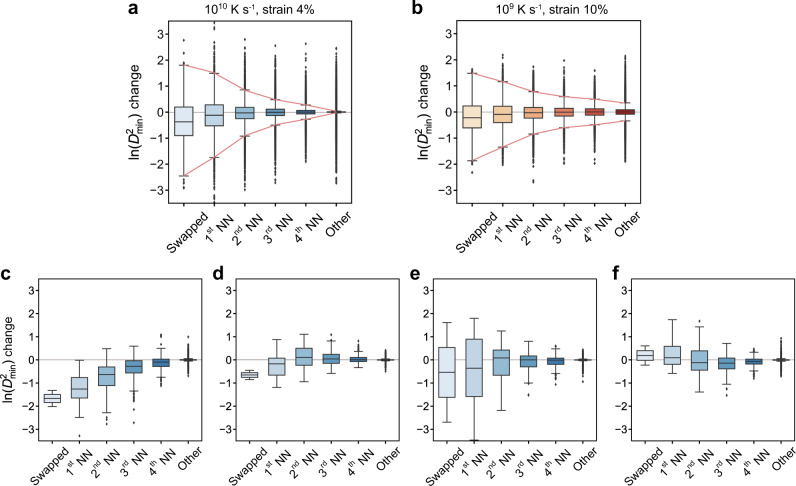
The strengthening mechanism. **a**, **b** Statistics of ln(${D}_{{{\min }}}^{2}$) change after each optimization step when using (**a**) GNN model for strain 4% to strengthen Cu_50_Zr_50_ – 10^10^ K s^−1^ and (**b**) GNN for strain 10% to strengthen Cu_50_Zr_50_ − 10^9^ K s^−1^. Atoms are grouped into six categories: the swapped atoms, 1st, 2nd, 3rd, and 4th neighbors of the swapped atoms, and all the other atoms. All the accepted steps are included in the statistics. In the box plots, ends of box spans from 25–75% percentile, black line in the box represents the median, whiskers show 1.5 times the inter-quartile range, and points outside the whiskers show outliers. **c**–**f** Typical ln(${D}_{{{\min }}}^{2}$) change in a single optimization step.

It is interesting to take a closer look at how the GNN-guided strengthening works. We extract the statistics of ln(${D}_{{{\min }}}^{2}$) change after each accepted optimization step (Fig. 4a, b). Atoms are grouped into six categories: the swapped atoms (i.e., two in each step), 1st, 2nd, 3rd, and 4th neighboring shells of the swapped atoms, and all the other atoms farther away. Results show that the optimization mainly focuses on the swapped atoms and their 1st neighbors; the farther from the swapped atoms, the weaker the optimization effect. Figure 4c–f further present some representative scenarios in a single optimization step: (i) the swapped atoms and their neighbor regions up to the 4th shell are all notably strengthened; (ii) the swapped atoms and their 1st neighbors are strengthened, while neighbors beyond the 1st shell are softened slightly; (iii) a compromise case where one swapped atom and some of the neighbors are strengthened, whereas the others are softened; iv) the swapped atoms and their 1st neighbors are softened yet the 2nd, 3rd, and 4th neighbors are strengthened. These scenarios have shown various interesting effects of the high-impact atoms (or atoms pairs) on strengthening. After accumulating the structural adjustments triggered by swaps of these high-impact atoms, the plastic resistance can be enhanced in a controllable way.

**Fig. 5:**
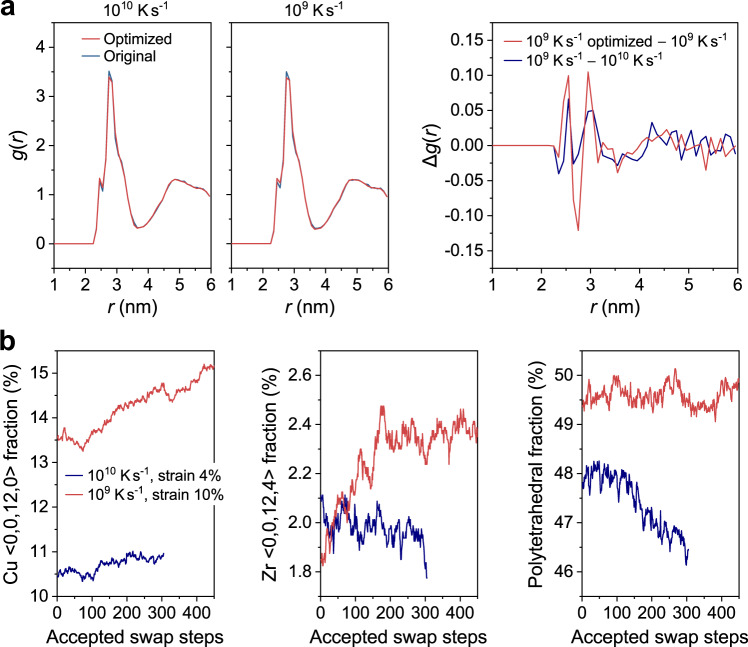
Structural evolution with optimization. **a** Pair correlation function, *g*(*r*), of the original Cu_50_Zr_50_ − 10^10^ K s^−1^ and 10^9^ K s^−1^ configurations and of the GNN-optimized configurations. Δ*g*(*r*), the difference of *g*(*r*) between two states, induced by the GNN-guided optimization and the decrease of quenching rate are derived and compared. **b** Tracking the fraction of typical Voronoi motifs, i.e., Cu-centered <0, 0, 12, 0> icosahedra, Zr-centered <0, 0, 12, 4> and polytetrahedral clusters, during the optimization process.

Given the success of the GNN-guided optimization, it is of interest to take a sip of the structural evolution during optimization. As a baseline, we first derive the pair correlation function, *g*(*r*), of the original and optimized states (Fig. 5a). We find the *g*(*r*) values before and after the optimization are overall similar. To examine the subtle changes of peak heights, we further extract the Δ*g*(*r*), the difference of *g*(*r*) between the two states of interest, *g*_1_(*r*)–*g*_2_(*r*). As the GNN-guided optimization as well as the decrease of quenching rate as a traditional protocol can both enhance the plastic resistance, we then extract the Δ*g*(*r*) caused by the two protocols, as a comparison (right panel in Fig. 5a). Interestingly, for the two cases, the ups and downs within *r* < 3.2 Å show some similar traits. This *r* regime is mainly dominated by Cu–Cu and Cu–Zr pairs, which suggests that the bonding between Cu–Cu and Cu–Zr may show some similar changes in distance. For larger *r* beyond 4.0 Å, which falls in the 2nd peak envelope of *g*(*r*), the change in the two cases is basically opposite (e.g., one is up and another is down). This may indicate a different mechanism in this *r* regime, which is dominated by MRO and beyond (more analysis will be conducted in future work).

As another analysis, we perform Voronoi tessellation analysis, a common structural analysis framework for glasses, to track the structural changes during optimization. Figure 5b monitors some typical Voronoi motifs that have long been proposed to be critical to glass stability^36^. During optimization, the fraction of Cu atoms centered by <0, 0, 12, 0> icosahedra increases by ~1.5% and ~0.5% for Cu_50_Zr_50_ – 10^10^ K s^−1^ and 10^9^ K s^−1^, respectively (Fig. 5b). This increase, though insignificant, seems to agree with the long-lived understanding of more icosahedra, more stable glass. However, the increase of icosahedra is far less prominent compared to the ln(${D}_{{{\min }}}^{2}$) change during optimization. For example, after 120 swaps, the ln(${D}_{{{\min }}}^{2}$) of Cu_50_Zr_50_ – 10^10^ K s^−1^ is already lower than that of Cu_50_Zr_50_ – 10^9^ K s^−1^ (Fig. 3b); however, the corresponding icosahedra fraction at that step is still ~3% lower than that of 10^9^ K s^−1^ (Fig. 5b). For the Zr atoms surrounded by <0, 0, 12, 4>, the fraction experiences a great oscillation and overall shows a small change, i.e., increases by ~0.6% and decreases by ~0.3% for Cu_50_Zr_50_ – 10^10^ K s^−1^ and 10^9^ K s^−1^, respectively (Fig. 5b). Similar oscillation is observed for the polytetrahedral clusters^36^ (with 2*n*_4_ + *n*_5_ = 12, where *n*_4_ and *n*_5_ are 4-fold and 5-fold Voronoi indices, Fig. 5b). These results indicate a weak correlation between plastic resistance and these representative clusters.

Resorting to the physical-inspired features may help us gain some intuitive understanding of the structural reasons for the improved plastic resistance, however, the connection is still largely empirical. We are working on clarifying the structural mechanism of strengthening by directly interpreting the GNN models, such as quantifying the neighbor contributions based on attention weights and mining the graphic traits of the high-impact atoms or substructures for plastic resistance. The aim is to make the GNN models less like a “black box”, so as to provide insightful details about the glass structure and its relationship with property.

## Discussion

This work demonstrates that the collaboration of GNN as decider and swap MC as sampler can open an avenue for the property-oriented inverse design of glass structure. To better achieve this goal, we also design several useful strategies to accelerate the optimization:

I. Instead of randomly selecting Cu and Zr atoms for the swap trial, we use the GNN-predicted plastic susceptibility to bias the selection. The probability for an atom *i* to be selected is,
  5 $${p}_{i}=\frac{{{{{{\rm{softmax}}}}}}({z}_{i})^n}{{\sum }_{{{{{{\rm{A}}}}}}}{{{{{\rm{softmax}}}}}}({z}_{i})^n}$$
  where *z*_*i*_ is the GNN-predicted ln(${D}_{{{\min }}}^{2}$) of atom *i*, A is the element type of atom *i*, and *n* is an exponential number controlling the inclination of selecting large ln(${D}_{{{\min }}}^{2}$) atoms (*n* is set as 6 in this work). In each trial, we will calculate *p*_*i*_ for each Cu and Zr atom, and sample one Cu and one Zr atom according to the probability distribution of each element type. This biased sampling allows us to “directionally strengthen” the soft spots without changing too much of the hard backbone.
II. We focus on sampling the inherent structures, namely the local minima in PES, to reduce the search space. In practice, after an atom pair swap, we apply the conjugate-gradient algorithm to get the inherent structure and let GNN decide whether to accept or not based on the inherent structure. This structural search approach is more of a molecular static method instead of a molecular dynamical one. In traditional swap MC simulations, whether to accept a swap trial is normally determined by the instant gain after swapping^12^; however, when the size difference of atoms is too large, the acceptance rate of swap trials will quickly diminish to ~0. By allowing the environments to modify to accommodate the swapped atoms, we can also make more feasible decisions of whether this swap is acceptable.
III. In each trial, we do not have to reconstruct the entire graph. After obtaining the inherent structure, our tests indicate that we only need to redefine the neighborhood for the hop-1 and hop-2 neighbors of the swapped atoms, and then reconstruct graphs for those atoms. After that, we will recalculate the properties of the atoms up to hop-*N* layer around those atoms with graph reconstructed, as the “affected zone” of graph reconstruction extends to hop-*N* neighbors. For small systems, such affected zone may easily propagate to the whole system, but this is very useful in the future study of large systems, helping to avoid repeated calculations.
IV. Finally, in our implementation, the entire swap trial and GNN evaluation procedure are executed in GPUs, so the computation speed is fast. The GNN takes significantly longer for training, but is much faster in prediction (even in milliseconds). The unprecedented parallel computation power of GPU greatly accelerates the optimization.

We note that the possible performance degradation of GNN during the optimization procedure is an issue that needs caution. This is because the distribution of the target variables (in essence, the atomic environments) can gradually get away from the training distribution. In this work, the change of Pearson coefficient on the accepted configurations is monitored in Supplementary Fig. 10. The Pearson coefficient decreases from 0.809 to 0.741 for Cu_50_Zr_50_ – 10^10^ K s^−1^, strain 4%, and from 0.851 to 0.821 for 10^9^ K s^−1^, strain 10%. While we conclude that the accuracy is still sufficient to support the optimization, and the optimization does continue to make progress (as seen in Fig. 3b and f), it would be of interest to incorporate ideas such as active learning to iteratively improve the GNN model. For example, one can set up some checkpoints to validate the performance on the accepted configurations, and if there is a large performance loss, the configurations can be added to the training set and retrain the GNN model.

It is also of interest to try using the true AQS simulations directly as decider, which could then form some upper bound for the optimization performance. Replacing the GNN estimation with a true MD simulation will undoubtedly lead to improved accuracy in decision making. Despite GNN has achieved high Pearson coefficients (Fig. 2), there are still discrepancies with the true MD simulation. This has caused fluctuations of $\langle {{{{\mathrm{ln}}}}}({D}_{{{\min }}}^{2})\rangle$ for the configurations accepted by GNN as decider (Fig. 3b and f), and $\langle {{{{\mathrm{ln}}}}}({D}_{{{\min }}}^{2})\rangle$ should monotonically decrease if we elaborately simulate the deformation of each trial configuration and then decide whether to accept or not. However, this is time-consuming. If we simulate twelve different modes of AQS deformation, the computation time (e.g., using Intel Xeon Gold 6126 CPU, 2.60 GHz) is roughly 100 CPU hours. But if we employ GNN to estimate, the prediction time (e.g., using NVDIA Tesla P100) is only 0.17 seconds. The time difference would be quite large if we do ~10000 rounds of trial (as Cu and Zr have a large size difference, the acceptance rate is relatively low). This makes GNN a reasonable and faster surrogate model for the simulations.

Furthermore, despite the prediction time of GNN is sufficiently short for the current glass system, the tradeoff between speed and accuracy is an issue worth noting. In certain cases, it can be possible to build much faster networks with a small decrease in accuracy, and the optimization can run more rounds in a fixed computing time. When designing the model infrastructure, especially when deciding between “massive” and “lightweight” models, issues such as the training time, the requirement of memory as well as the inclination of overfitting should also be taken into consideration. Currently, it is hard to figure out where the optimal tradeoff lies, which requires further studies in this direction.

Taken together, this work forms a first attempt to introduce artificial intelligence methods to tackle the inverse design problem in the glass domain. This is achieved by pairing a cutting-edge deep learning framework, GNN, as a surrogate model, with a tailored structural search method modified from swap MC. The high accuracy of our GNN model and massively parallel computing power of GPU solves the most time-consuming part of the structural search, significantly reducing the time for evaluating the numerous glass candidates. The protocol proposed here paves a new way for the inverse design of glass structures with desired functionalities. In future studies, we will explore more optimization algorithms, such as evolutionary algorithms, to see if a global optimal structure could be obtained efficiently. We also plan to try generator networks, based on conditional variational autoencoders or generative adversarial networks, to test whether they can reliably propose promising structures in “one-shot”. It is also interesting to combine multiple glass properties into a multi-objective optimization task. In addition, the current design case is at a fixed stoichiometry, we can also explore to allow the stoichiometry to vary, thereby searching over a larger compositional space. Overall, with the rigorous development of artificial intelligence in recent years, data-driven methods and optimization algorithms can now be integrated to form a toolbox, revealing the rules behind complex phenomena. Applying this toolbox to glass research, we can deepen the understanding of key scientific issues such as the structure-property correlation, and provide a theoretical basis for the controllable regulation of glass properties.

## Methods

### Glass samples preparation

Molecular dynamics (MD) simulations using LAMMPS^38^ are conducted to prepare the Cu–Zr glass configurations, using a set of embedded-atom-method (EAM) potentials^39^. Cu_64_Zr_36_ and Cu_50_Zr_50_ samples, each containing 16,000 atoms, are quenched to 0 K from the melts. The quenching is performed at a rate of 10^9^ or 10^10^ K s^−1^, using a Nose-Hoover thermostat with zero external pressure. Periodic boundary conditions (PBC) are applied in all three directions during MD simulation. The timestep is 1 fs.

### AQS data generation

For each glass configuration, we stimulate twelve AQS^37^ loading conditions, i.e., uniaxial tension and compression along x, y, and z-direction (6 conditions) and simple shear along xy+, xy−, yz+, yz−, xz+ and xz− (6 conditions). On each deformation step, an affine strain of 10^−4^ is imposed along the loading direction, followed by energy minimization using the conjugate-gradient method. The simulations are conducted using LAMMPS^38^ and periodic boundary conditions (PBC) were applied in all three directions. More complex deformation modes such as pure shear are not included, as the twelve modes can already reflect a comprehensive plastic resistance of each atom, while the more complex mode could be considered as a superposition of the elementary modes.

The plastic indicator, namely the natural logarithm of non-affine displacement (${D}_{{{\min }}}^{2}$)^40^, is calculated for the strains from 0.5% to 14%, to quantify the deformation propensity of atoms at each strain. The use of ln(${D}_{{{\min }}}^{2}$) as the plastic indicator is to convert the “long-tail”, lognormal-like distribution of ${D}_{{{\min }}}^{2}$ to a normal-like distribution, for ease of training.

### Swap Monte Carlo

Swap MC has been proposed as a powerful method to search the configuration space of disordered materials^12^. In traditional swap MC simulations, two types of trials are conducted, (i) atom pair swap and (ii) displacement move. The Metropolis–Hastings criterion^41^, based on the energy difference and temperature, is usually used to decide whether a certain trial is acceptable or not,

6 $$p=\left\{\begin{array}{cc}1 & {{{{{\rm{if}}}}}}\,\Delta {{{E}}}\,\le \,0\\ {e}^{-\frac{\varDelta E}{{{{kT}}}}} & {{{{{\rm{if}}}}}}\,\Delta {{{E}}}\, > \,0\end{array}\right.$$

where *p* is the acceptance probability, Δ*E* is the energy difference between the trial state and old state, *E*^trial^-*E*^old^ ; *k* is the Boltzmann constant and *T* is the simulation temperature.

### GNN-guided, property-oriented swap Monte Carlo

In this work, we let the GNN predictions replace the role of energy (*E*) in Eq. 6 and design a few rules to decide whether to accept a trial (as illustrated in Fig. 3a). The procedure of the GNN-guided, property-oriented swap MC is as follows,

(i) Apply GNN to predict the plastic metric, ln(${D}_{{{\min }}}^{2}$), of each atom in the starting configuration, and record the initial values of the designed target functions. The right panel in Fig. 3a shows the seven target functions (the maximum, mean, and five quantiles) used in this work;

(ii) Select a pair of atoms of different species according to probabilities based on ln(${D}_{{{\min }}}^{2}$) (see point (i) in Discussion and Eq. 5 for more details) and swap their species. Then obtain the inherent structure, i.e., the local minimum in PES, using the conjugate-gradient method (see point (ii) in Discussion for the reason why we focus on inherent structures only). The volume is kept fixed to avoid the volumetric effect on plastic resistance. Reconstruct the graph and update  the predicted ln(${D}_{{{\min }}}^{2}$) for those atoms that are affected by the swap trial (see point (iii) in Discussion for details). Afterwards, for each of the seven target functions, calculate its change after the trial to determine its associated acceptance probability,

7 $$p=\left\{\begin{array}{cc}1 & {{{{{\rm{if}}}}}}\,\Delta {{{F}}}\,\le \,0\\ {e}^{-\frac{\Delta {{{F}}}}{C}} & {{{{{\rm{if}}}}}}\,\Delta {{{F}}}\, > \,0\end{array}\right.$$

where Δ*F* is the target function difference between the trial state and old state, *F*^trial^-*F*^old^, and *C* is a constant dependent on the specific target function for tuning the acceptance probability. The ultimate acceptance probability is defined as ∏*p*, where *p* is the probability associated with each target function and ∏ denotes the sequence product. If accepted, the trial configuration will be set as the starting configuration for the next swap trial; and if rejected, the configuration will not change.

(iii) Repeat the (i) and (ii) steps for a fixed number of rounds, until being close to the target property.

## Supplementary information

Supplementary information

## Data Availability

All data needed to evaluate the conclusions in the paper are present in the paper and/or the Supplementary Materials. The raw data used in this work are available from the corresponding author upon reasonable request.

## Code availability

The Pytorch implementation of our GNN is available at https://github.com/Qi-max/gnn-for-glass.
