# Supplementary material for: Inverse design of glass structure with deep graph neural networks
Source: Nat Commun. 2021 Sep 9;12:5359. doi: 10.1038/s41467-021-25490-x (PMC8429760; doi:10.1038/s41467-021-25490-x)
Supplement: Supplementary file 1 — Supplementary information [file 41467_2021_25490_MOESM1_ESM.pdf]

Supplementary Information

Inverse design of glass structure with deep graph neural networks

Qi Wang[1 *], Longfei Zhang[2]

[1] Science and Technology on Surface Physics and Chemistry Laboratory, P. O. Box 9-35, Jiangyou, Sichuan 621908, China

[2] School of Software, Beihang University, Beijing 100191, China

*Corresponding author. Email: qwang_mse@163.com

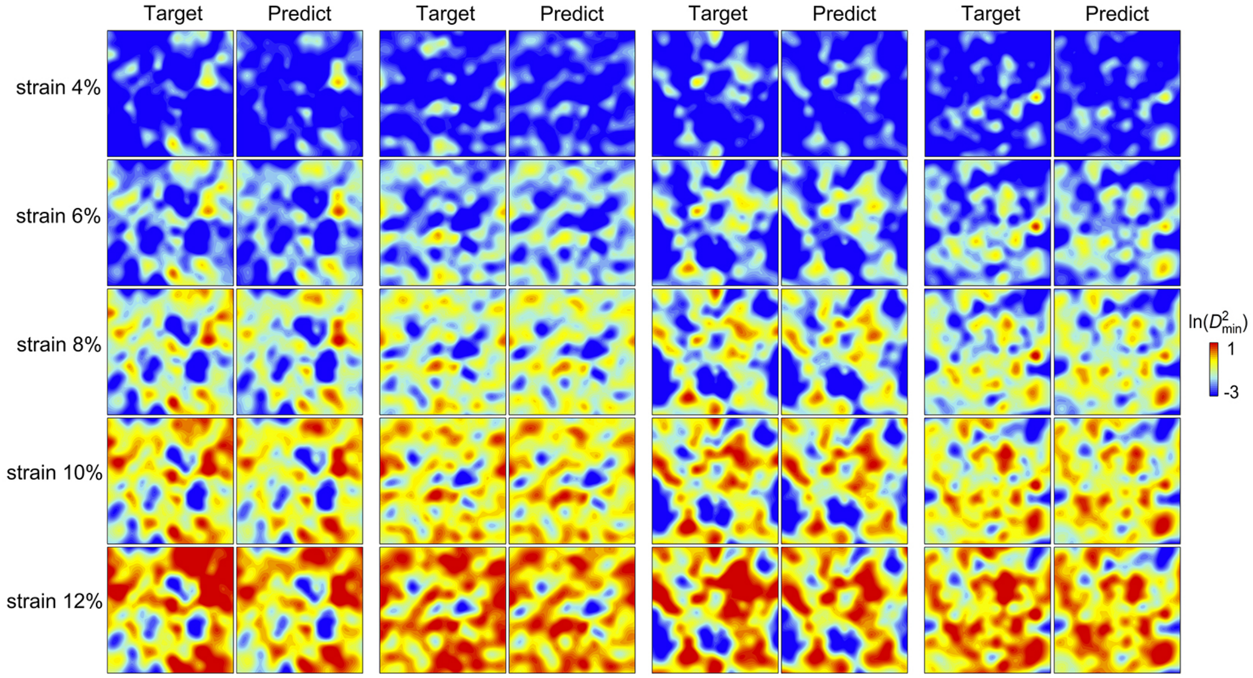

Supplementary Figure 1. Target versus GNN predicted ln(D^2_{min}) in typical slices of 3 Å thickness in a $Cu_{64}Zr_{36}$ – 10^9 K s^{-1} configuration at strains of 4%, 6%, 8%, 10% and 12%. The GNN model trained and verified in $Cu_{64}Zr_{36}$ – 10^{10} K s^{-1} at each strain is used for prediction (generalization from $Cu_{64}Zr_{36}$ – 10^{10} K s^{-1} to $Cu_{64}Zr_{36}$ – 10^9 K s^{-1}).

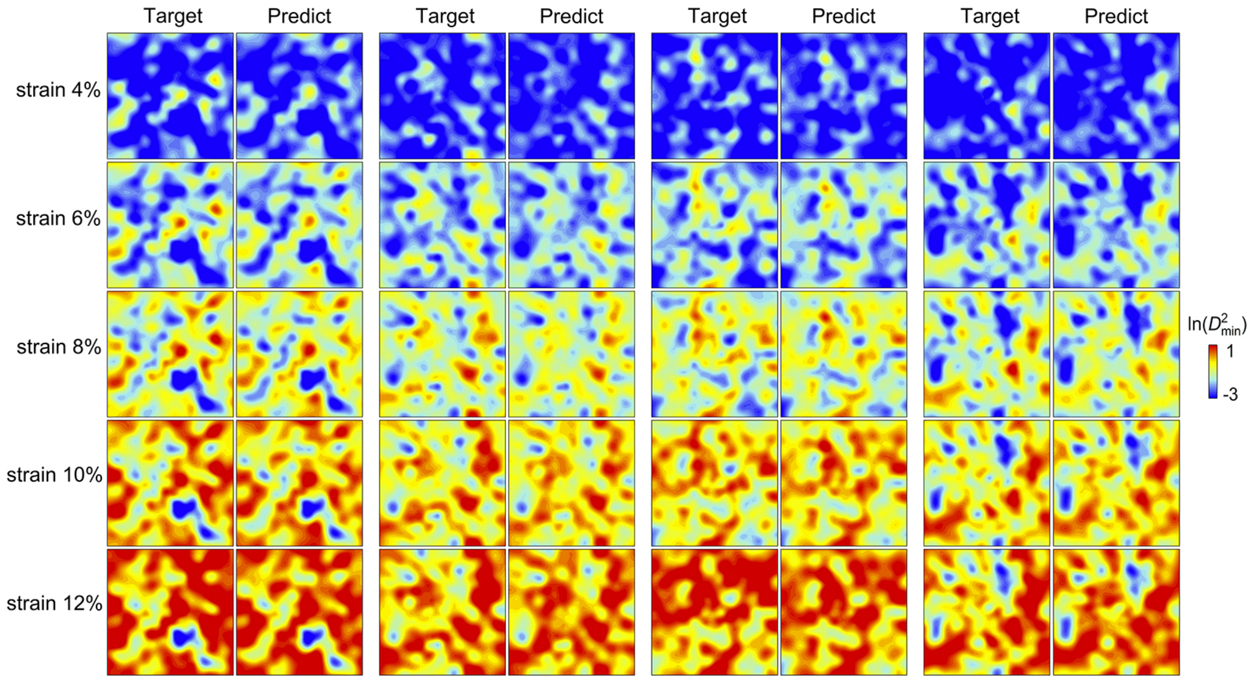

Supplementary Figure 2. Target versus GNN predicted ln(D^2_{min}) in typical slices of 3 Å thickness of a $Cu_{64}Zr_{36}$ – 10^{10} K s^{-1} configuration at strains of 4%, 6%, 8%, 10% and 12%.

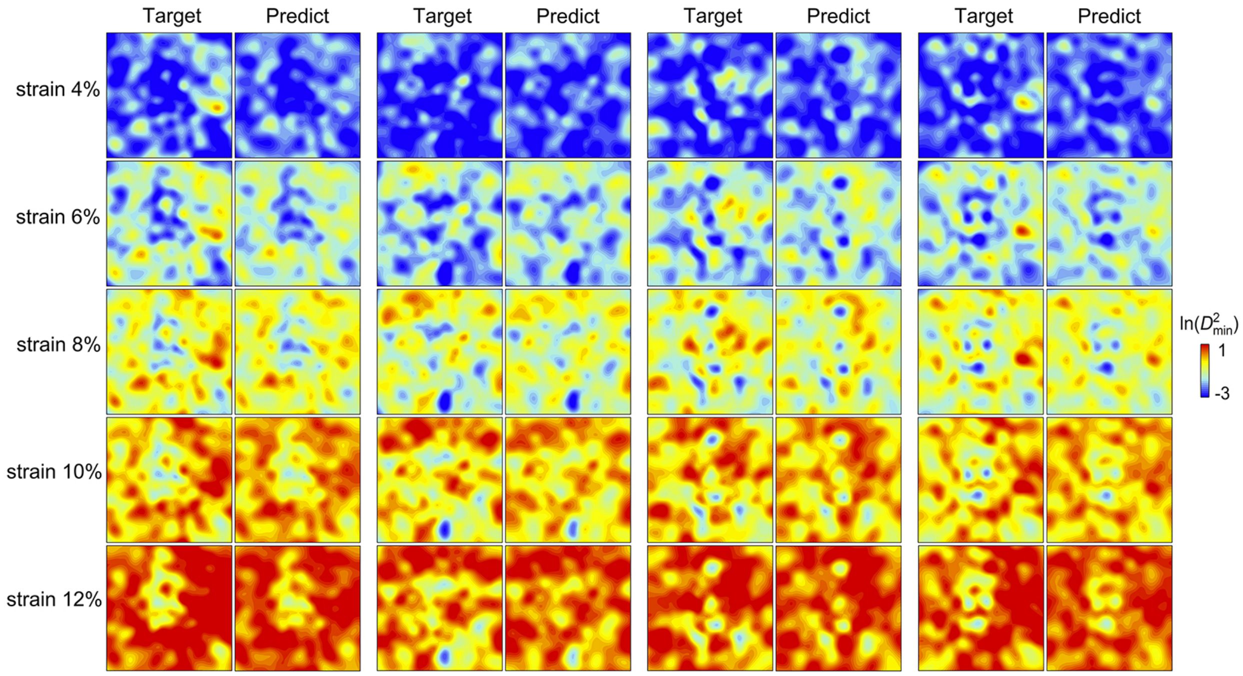

Supplementary Figure 3. Target versus GNN predicted ln(D^2_{min}) in typical slices of 3 Å thickness of a $Cu_{50}Zr_{50}$ – 10^9 K s^{-1} configuration at strains of 4%, 6%, 8%, 10% and 12%. The GNN model trained and verified in $Cu_{50}Zr_{50}$ – 10^{10} K s^{-1} at each strain is used for prediction (generalization from $Cu_{50}Zr_{50}$ – 10^{10} K s^{-1} to $Cu_{50}Zr_{50}$ – 10^9 K s^{-1}).

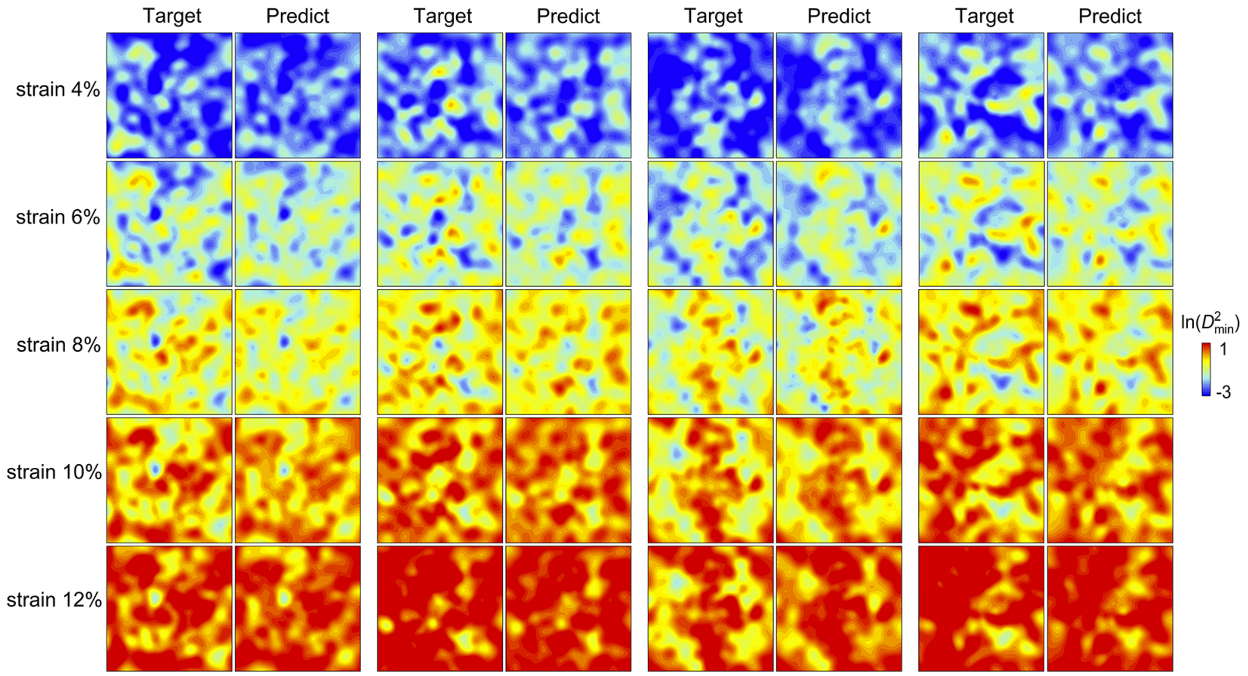

Supplementary Figure 4. Target versus GNN predicted ln(D^2_{min}) in typical slices of 3 Å thickness of a $Cu_{50}Zr_{50}$ – 10^{10} K s^{-1} configuration at strains of 4%, 6%, 8%, 10% and 12%.

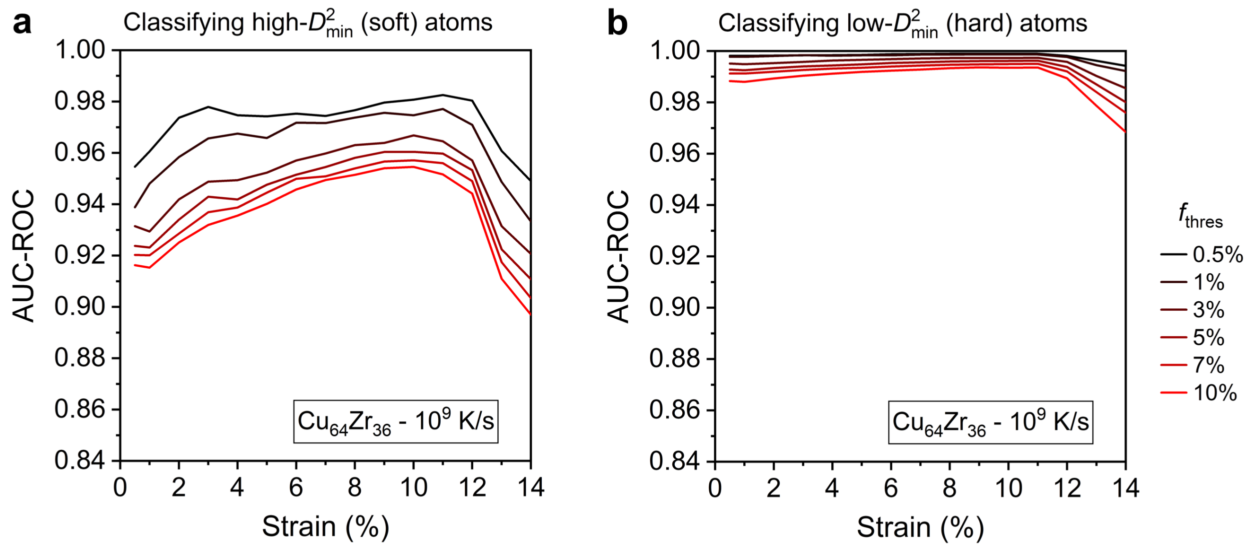

Supplementary Figure 5. Classifying the (**a**) soft and (**b**) hard atoms in $Cu_{64}Zr_{36}$ – 10^9 K s^{-1} test configurations using the GNN predicted $\ln(D^2_{min})$. The area under receiver operating characteristic curve (AUC-ROC) is derived as the classification metric (an AUC-ROC of 1.0 indicates perfect classification, 0.5 indicates random chance level). A series of fraction threshold, f_{thres}, are employed to setup the classification task (f_{thres} = 0.5%, 1%, 3%, 7% and 10%), that is, atoms with D^2_{min} among the highest (lowest) f_{thres} at each strain will be designated as soft (hard) atoms, namely the positive class, and the remaining atoms will be the negative class.

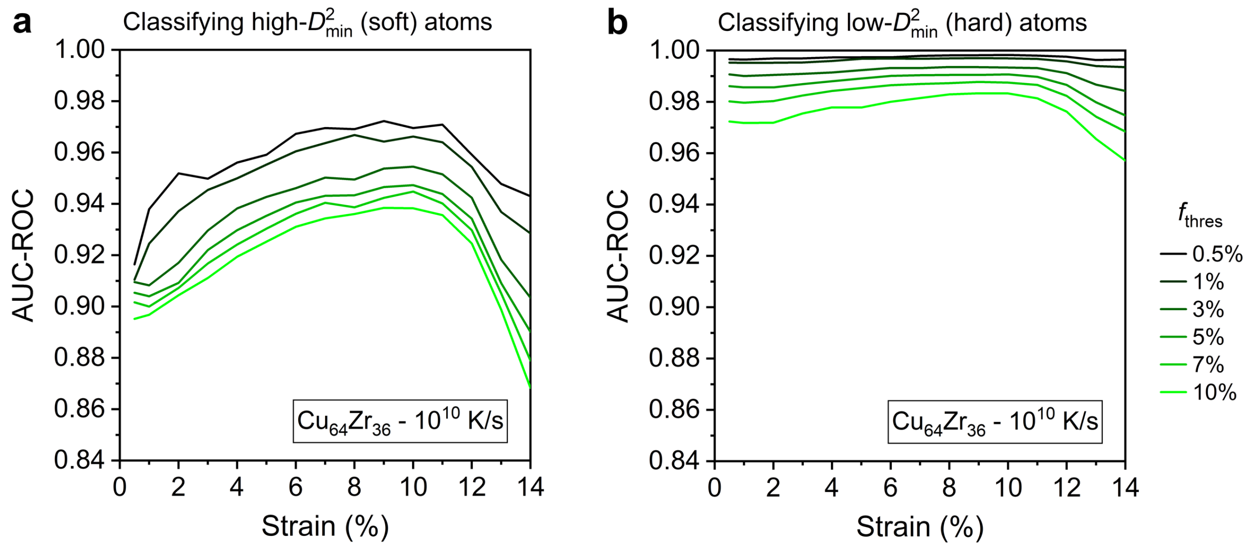

Supplementary Figure 6. Classifying the (**a**) soft and (**b**) hard atoms in $Cu_{64}Zr_{36}$ – 10^{10} K s^{-1} test configurations using the GNN predicted $\ln(D^2_{min})$. The classification settings are the same as that described in Fig. S5.

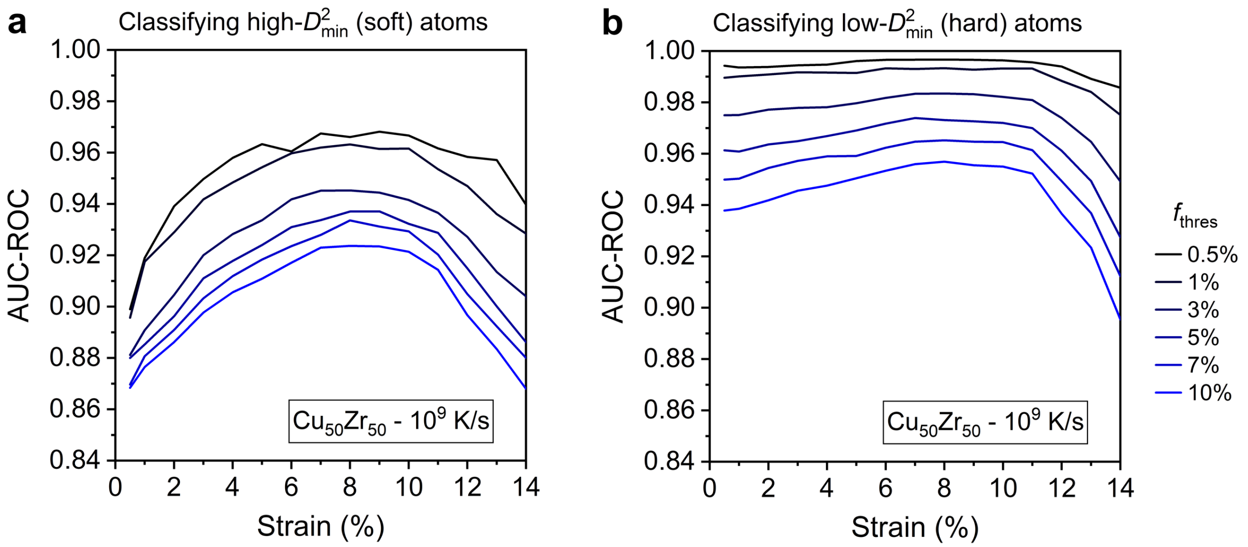

Supplementary Figure 7. Classifying the (**a**) soft and (**b**) hard atoms in $Cu_{50}Zr_{50}$ – 10^9 K s^{-1} test configurations using the GNN predicted $\ln(D^2_{min})$. The classification settings are the same as that described in Fig. S5.

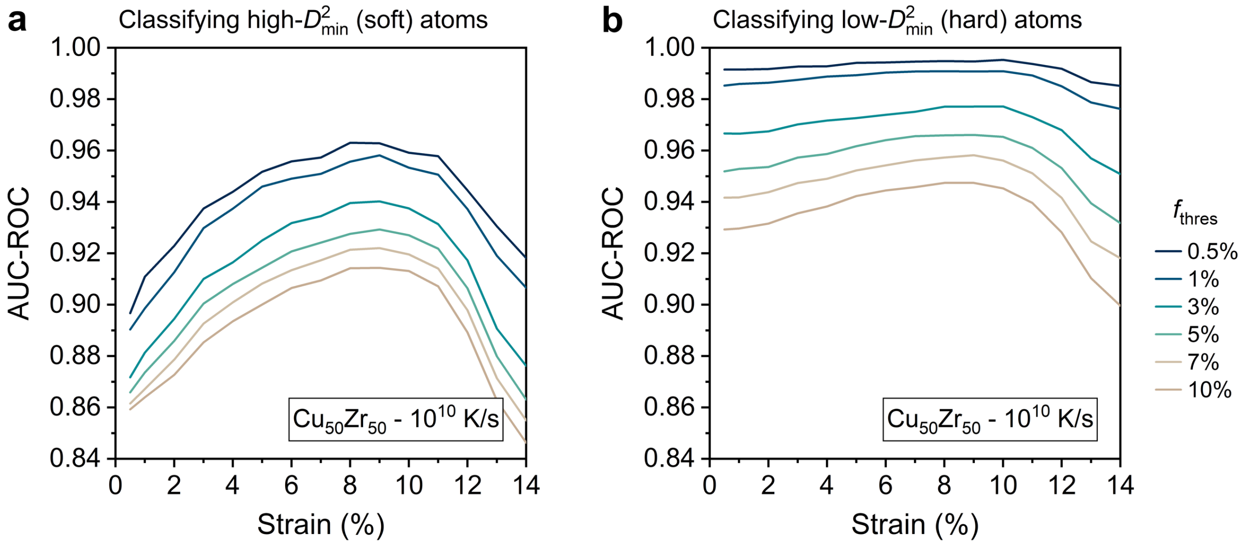

Supplementary Figure 8. Classifying the (**a**) soft and (**b**) hard atoms in $Cu_{50}Zr_{50}$ – 10^{10} K s^{-1} test configurations using the GNN predicted $\ln(D^2_{min})$. The classification settings are the same as that described in Fig. S5.

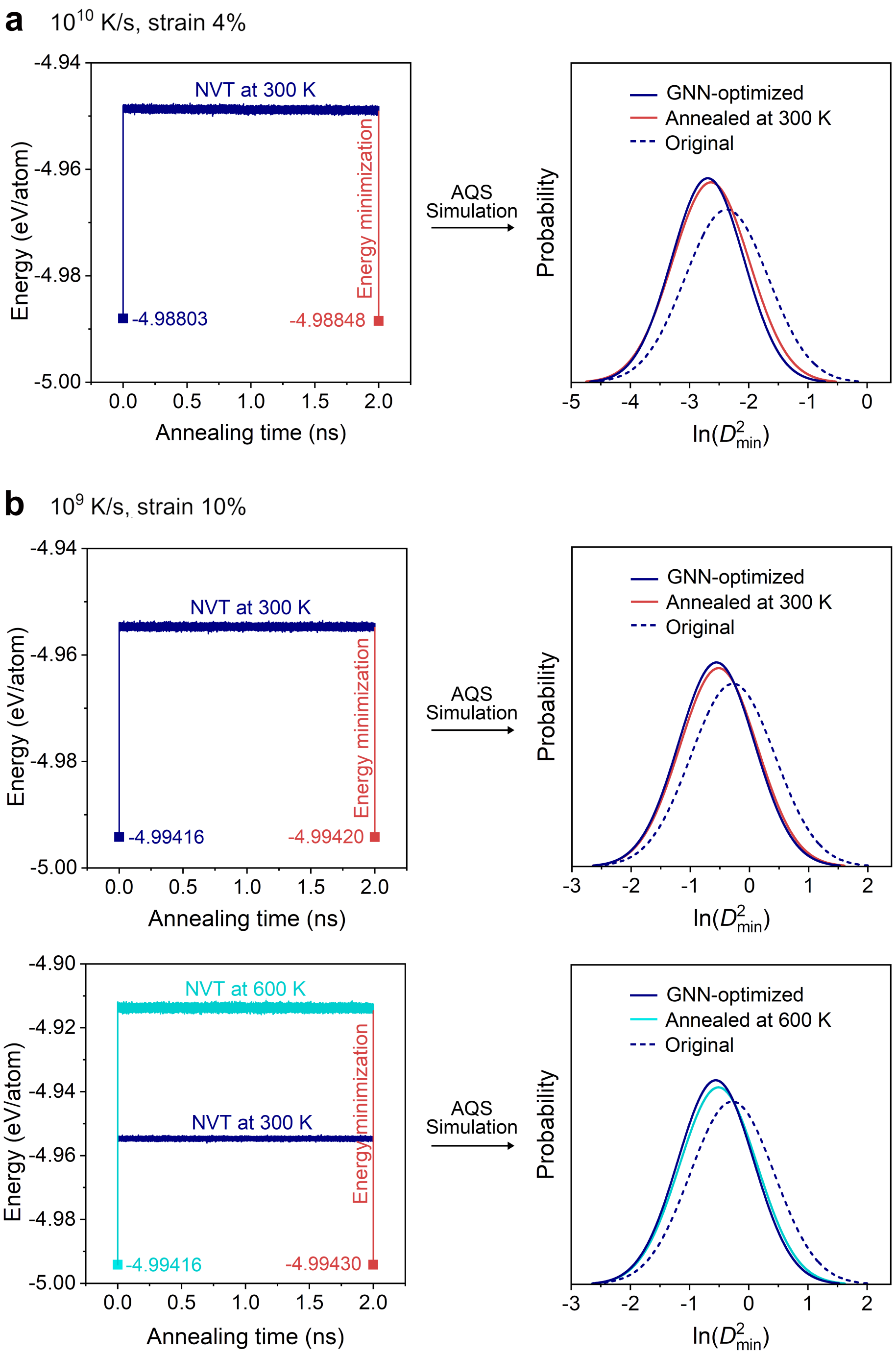

Supplementary Figure 9. (**a**) Annealing the GNN-optimized $Cu_{50}Zr_{50}$ – 10^{10} K s^{-1} configuration under NVT ensemble at 300 K for 2 ns and get the inherent structure by energy minimization (left panel). Distribution of $\ln(D^2_{\mathrm{min}})$ of the GNN-optimized configuration, the GNN-optimized configuration after annealing at 300 K, and the original unoptimized configuration, respectively (right). (**b**) Annealing the GNN-optimized $Cu_{50}Zr_{50}$ – 10^9 K s^{-1} configuration under NVT ensemble at 300 K or 600 K for 2 ns and get the inherent structure by energy minimization (left). Distribution of $\ln(D^2_{\mathrm{min}})$ of the GNN-optimized configuration, the GNN-optimized configuration after annealing at 300 K or 600 K and the original unoptimized configuration, respectively (right).

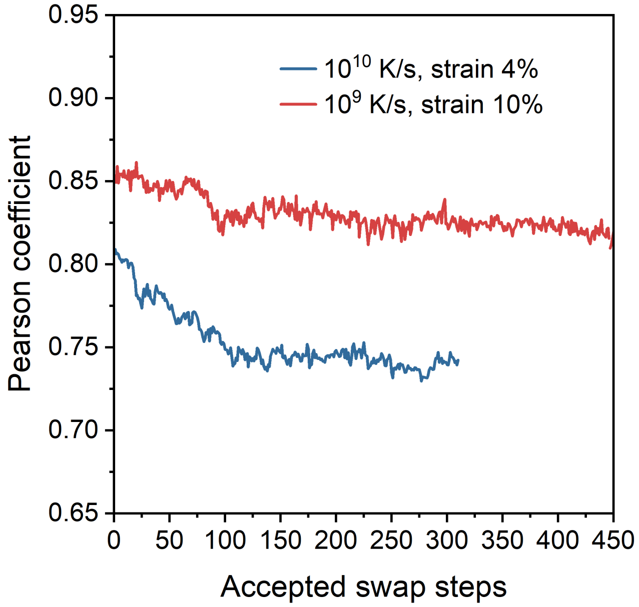

Supplementary Figure 10. The change of Pearson coefficient with the progress of optimization.
